# Probabilistic Multi-Sensor Fusion Based Indoor Positioning System on a Mobile Device

**DOI:** 10.3390/s151229867

**Published:** 2015-12-14

**Authors:** Xiang He, Daniel N. Aloi, Jia Li

**Affiliations:** Department of Electrical and Computer Engineering, Oakland University, 2200 N Squirrel Road, Rochester, MI 48309, USA; xhe2@oakland.edu (X.H.); aloi@oakland.edu (D.A.)

**Keywords:** indoor positioning, HMM framework, graph structure, multimodal particle filter, sensor fusion, iOS platform

## Abstract

Nowadays, smart mobile devices include more and more sensors on board, such as motion sensors (accelerometer, gyroscope, magnetometer), wireless signal strength indicators (WiFi, Bluetooth, Zigbee), and visual sensors (LiDAR, camera). People have developed various indoor positioning techniques based on these sensors. In this paper, the probabilistic fusion of multiple sensors is investigated in a hidden Markov model (HMM) framework for mobile-device user-positioning. We propose a graph structure to store the model constructed by multiple sensors during the offline training phase, and a multimodal particle filter to seamlessly fuse the information during the online tracking phase. Based on our algorithm, we develop an indoor positioning system on the iOS platform. The experiments carried out in a typical indoor environment have shown promising results for our proposed algorithm and system design.

## 1. Introduction

In recent years, researchers have developed various approaches for mobile-device (smartphone, tablet) user-positioning in GPS-denied indoor environment. To name a few, radio frequency (RF) fingerprinting techniques, motion-sensor-based pedestrian dead reckoning (PDR) techniques, and visual-sensor-based feature matching techniques, are some of the most popular approaches in indoor positioning. However, all of them have their own limitations. RF fingerprinting techniques (Bluetooth [1], RFID [2], Zigbee [3] and WiFi [4,5,6],) have the problem of signal fluctuation due to the multipath fading effect in indoor environment. The motion-sensor-based PDR approach [7,8] suffers from the fact that the motion sensors equipped in the mobile device are low cost Micro Electromechanical System (MEMS) sensors, which have relatively low accuracy. Thus, the integration drift will cause the positioning error to accumulate over time. The visual-sensor-based positioning techniques [9,10,11,12,13] extract features (SIFT [14], SURF [15]) from captured images and compare them with an image database. These techniques are limited by their costly feature-matching algorithm and restricted computation resources on a mobile platform. In ASSIST [16], acoustic signals emitted from smartphone speakers are adopted to locate the user using the time difference of arrival (TDoA) multilateration method. ASSIST can locate the user within 30 cm. However, this method requires sound receivers to be preinstalled in ceilings or walls, which adds extra infrastructure to the indoor environment.

To overcome the drawback of each sensor, people have come up with fusion approaches to combine different sensors to achieve a better positioning result. However, since the sensors are measuring different physical phenomena, it is not an easy task to effectively fuse the information from multiple sensors. The existing sensor fusion approaches for positioning involve decision-level fusion and feature-level fusion. Decision-level fusion usually contains multiple local detectors and a fusion center. The local decisions are transmitted to the fusion center where the global decision is derived. The optimum decision rule under the Neyman-Pearson sense can be expressed as a function of the correlation coefficients of the local detectors. It has been shown that the performance of such distributed detection systems degrade as the degree of correlation increases [17]. This approach is easy to implement and computationally efficient. It has been widely used in wireless sensor networks (WSN) and some other research fields [18]. However, it is not practical in an indoor positioning system with multiple sensors due to the difficulty of determining the correlation coefficients between different sensors. On the other hand, feature-level fusion [19] is a more delicate fusion approach that extracts features from multiple sensor observations, and uses these features to represent the real world and help positioning. The problem with feature-level sensor fusion is the highly redundant sensor data in feature extraction. As there are multiple sensors, each sensor delivers different data about the surrounding environment; we have to determine an effective approach to extract the information and store it in an efficient way so that we can easily access them for the purpose of positioning. Existing fusion algorithms include Bayesian filtering techniques, such as the Kalman filter [20,21] and particle filter [22,23], and non-Bayesian filtering technique, like conditional random fields [24,25] and Dempster-Shafer theory [26,27]. Originally, the Kalman filter and particle filter are designed for state estimation in single-sensor measurements. However, information fusion, based on Bayesian filtering theory, has been studied and widely applied to multi-sensor systems. Generally, there are two types of methods used to process the measured sensor data. The first one is the centralized filter, where all sensor data are transferred to a fusion center for processing. The second one is the decentralized filter, where the information from local estimators can achieve the global optimal or suboptimal state estimate according to certain information fusion criterion.

In previous work [28], we adapted Gaussian process modeling of WiFi RF fingerprinting and a particle filter based localizer to a mobile device. Later on, we introduce motion sensors on board to inprove the positioning accuracy [29]. The algorithm is implemented on the iOS platform and tested in an indoor environment. In this paper, to further improve the positioning accuracy, we introduce visual sensors into our system. The probabilistic model for multi-sensor fusion is investigated in a hidden Markov model (HMM) framework, where the state transition model is defined as the user motion model, and the observation model includes a WiFi sensor model, camera sensor model, and motion sensor model. Researchers have applied HMM successfully in a WSN area. Huang *et al.* modeled the dynamic quantization and rate allocation in a sensor network with a fusion center as a finite state Markov chain, and designed an optimal quantizer using a stochastic control approach for state estimation in a hidden Markov model [30]. Rossi *et al.* developed a HMM framework that exploits time-correlation of the unknown binary source under observation through a WSN-reporting local sensor detection to a fusion center over Rayleigh fading channel interference [31]. To solve the HMM state estimation problem with multiple sensors, Blom *et al.* proposed the interacting multiple model (IMM) algorithm, which combines state hypotheses from multiple filter models to get a better state estimate of targets with changing dynamics [32]. The filter models used to form each state hypothesis can be derived to match the targets of interest’s behavior. In this paper, we propose a multimodal particle filter to seamlessly fuse the data from multiple sensors for HMM state estimation.

A graph structure G=(V,E) is developed to store the information effectively. The key idea is to represent the indoor environment using graph of which vertices correspond to segments of the indoor environment. The segments are predefined in an offline built 3D model. The vertices play an important role in the motion model, since they relate to movement choices, which are positions where the user has a limited amount of choices as to where to move next. The edges correspond to connections between different segments, which act as constraints of the user movement to reduce computation during the online tracking phase.

Specifically, we make the following contributions: Under the HMM framework, we propose a graph structure to store the model constructed by multiple sensors in the offline training phase, and a multimodal particle filter to efficiently fuse the information during the online tracking phase. The particle filter is able to handle the motion sensor drift problem during the resampling step. The WiFi signal strength fluctuation problem is mitigated using the motion sensor information to guide the particle propagation towards the higher likelihood field. Based on our algorithm, we develop an indoor positioning system on the iOS platform. To the best of our knowledge, our iOS application is the first one to achieve accurate, robust, and highly integrated indoor positioning by seamlessly fusing the information from the multiple sensors on board.

This paper is organized as follows: In the next section, we describe, in detail, the offline training phase. Then, in Section 3, a HMM framework is introduced to describe the probabilistic multi-sensor fusion. In Section 4, we talk about particle filter steps for HMM state estimation. The implementation on the iOS platform, and experimental results, are presented in Section 5. Finally, we conclude our research in Section 6 with a discussion of future work.

## 2. Offline Training Phase

In this section, we will discuss every detail of our preparation for online tracking.

### 2.1. 3D Modeling of Indoor Environments

**Figure 1:**
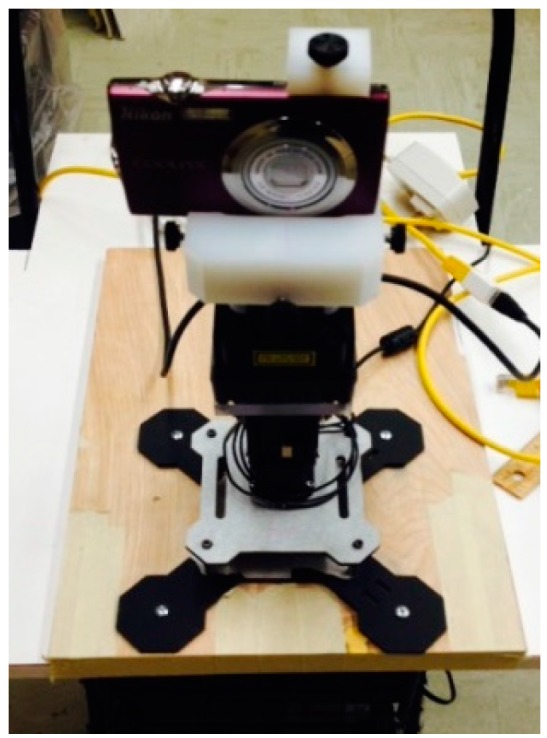
Snapshot of the LiDAR-camera scanning system.

A detailed 3D model of the indoor environment is constructed by fusing the data from a camera and a 2D line-scan LiDAR. Both devices are mounted rigidly on a robotic servo, which sweeps vertically to cover the third dimension (Figure 1). Fiducial target-based extrinsic calibration [33,34,35] is applied to acquire transformation matrices between LiDAR and the camera. Based on the transformation matrix, we perform registration to fuse the color images from the camera with the 3D point cloud from the LiDAR.

**Figure 2:**
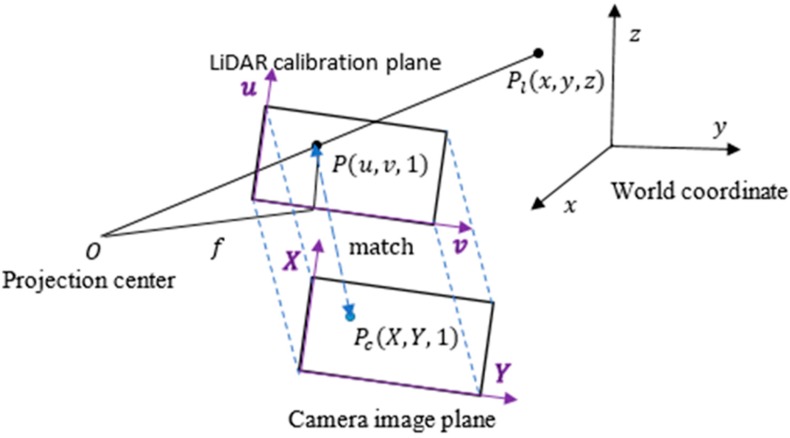
Pinhole camera model.

As shown in Figure 2, a 3D point in the LiDAR calibration plane is represented as $P_{l}={\left[x,y,z\right]}^{T}$ and its related pixel in the camera image plane is described as $P_{c}={\left[X,Y,1\right]}^{T}$. The 3D point $P_{l}$ with intensity information is projected to a calibration plane under a pinhole camera model. The calibration plane is defined at z=f and the projected point in the calibration plane is shown as $P={\left[u,v,1\right]}^{T}$. Based on similar triangle rules, we have the following relationship:

(1) $$u=f\frac{x}{z};v=f\frac{y}{z}$$

where f is the focal length of the camera. In order to fuse the information from LiDAR and the camera, we need to look for the relationship to match P and $P_{c}$.

**Figure 3:**
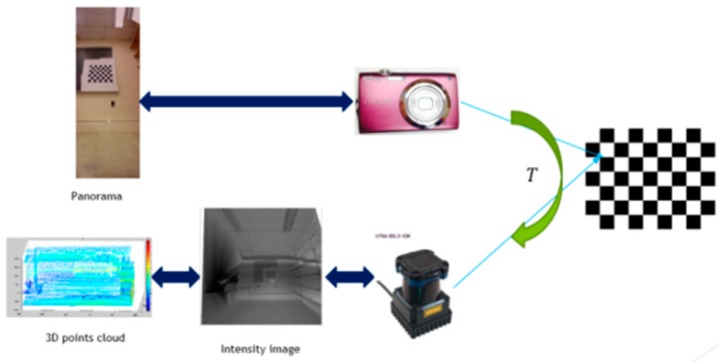
Extrinsic calibration procedure.

Figure 3 gives a workflow of the extrinsic calibration procedure. After projecting the 3D points to the calibration plane, we get a 2D point cloud. These 2D points are interpolated to generate a LiDAR intensity image. The problem of extrinsic calibration has become how to find the geometric constraints between a LiDAR intensity image and a camera image using the checkerboard pattern. The transformation of the checkerboard pattern from the LiDAR calibration coordinate frame to the camera coordinate frame is represented by a rigid 3 x 3 transformation matrix T.

(2) $$P_{c}=TP$$

**Figure 4:**
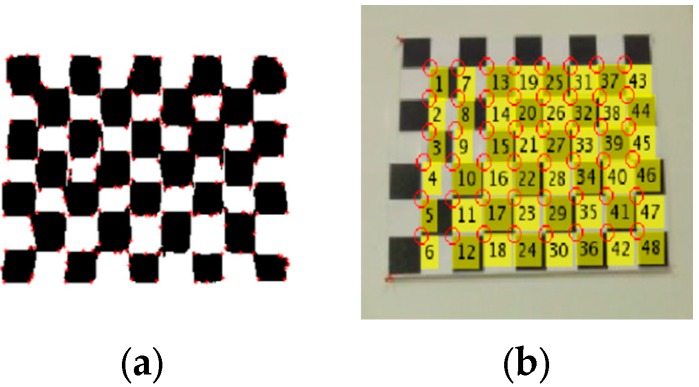
(**a**) Region of Interest (ROI) of LiDAR intensity image; (**b**) ROI of camera panorama.

As shown in Figure 4, to obtain the features of a checkerboard accurately, we select a Region of Interest (ROI) from the LiDAR intensity image and the camera panorama for the checkerboard pattern. Next, we take advantage of Random Sample Consensus (RANSAC) algorithm to find the correspondences between the LiDAR intensity images and the camera panorama images. In RANSAC, a pair of points is selected as an inlier only when the distance between them falls within the specified threshold. The distance metric used in RANSAC is as follows:

(3) $$D=\sum_{i=1}^{N}\min(d(P_{c},T(P)),\xi )$$

where P is a point in the LiDAR intensity image, $P_{c}$ is a point in the camera panorama image, T(P) is the projection of a point on the intensity image based on the transformation matrix T, d is the distance between a pair of points, ξ is the threshold, and N is the number of points.

The algorithm for generating the transformation matrix is summarized below:

(1) Find the inliers for the corners of checkerboard based on RANSAC algorithm.
  The RANSAC algorithm follows these steps:
  (a) Randomly select three pairs of points from the LiDAR intensity image and camera image to estimate a fitting model.
  (b) Calculate the transformation matrix T from the selected points.
  (c) Change the T value, if the distance matrix of a new T is less than the original one.
(2) Choose the transformation matrix T, which has the maximum inliers.
(3) Use all inlier point pairs to compute a refined transformation matrix T.

After generating the transformation matrices, we are able to stitch the camera panoramas together and fuse them with one LiDAR intensity image by applying the transformation matrices. Then, we back project the textured 2D points to a 3D color point cloud.

**Figure 5:**
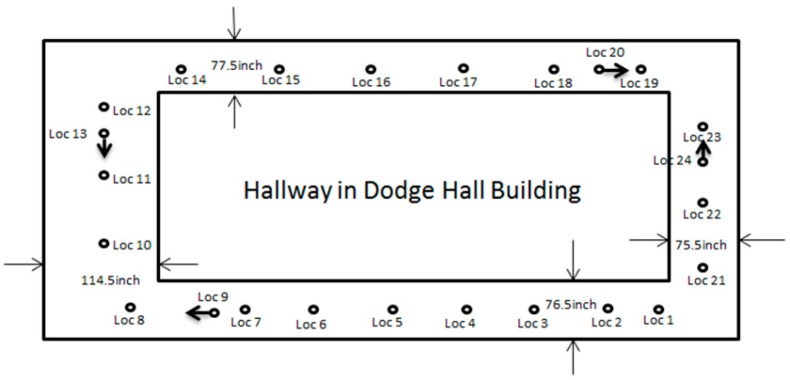
2D map of a corridor.

**Figure 6:**
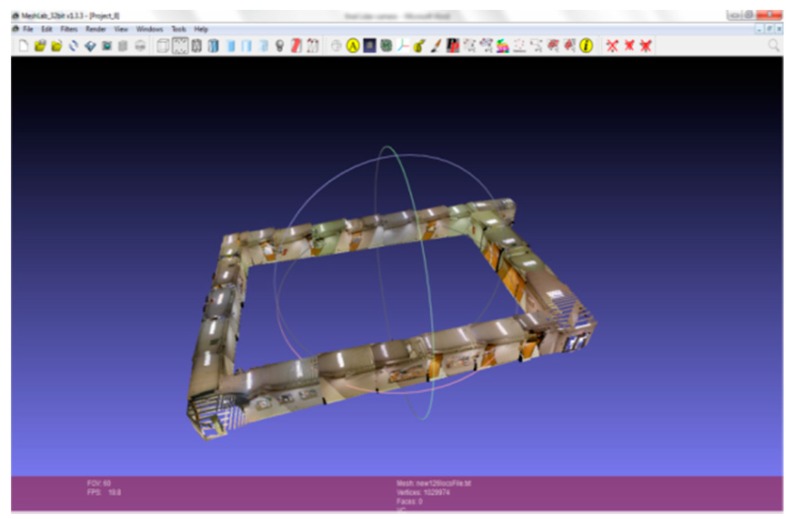
3D model of a corridor.

The extrinsic calibration result is applied to a large indoor environment. The LiDAR-camera scan system is mounted on a pushcart in order to record the data in stop-and-go mode. By manually aligning the data in each survey point, we can get a detailed 3D model of the indoor environment. At the same time, the 3D model is partitioned, based on the survey point locations. Figure 5 shows a 2D map of a large corridor area and survey point locations. The corresponding 3D model is shown in Figure 6. In order to construct a 3D model of the corridor, with an area of 630,000 square feet, we have collected 1,029,974 data points; each point with an XYZ and RGB value. Based on the high accuracy of the laser beam, this model is much more accurate than the 3D model generated from a RGB-D sensor. Admittedly, it is computational heavy to process the data in the offline training phase to build a detailed 3D color point cloud. However, during the online tracking phase, we only need to match the captured image with a local model instead of the entire one, which significantly reduces the cost.

### 2.2. Graph Structure Construction

**Figure 7:**
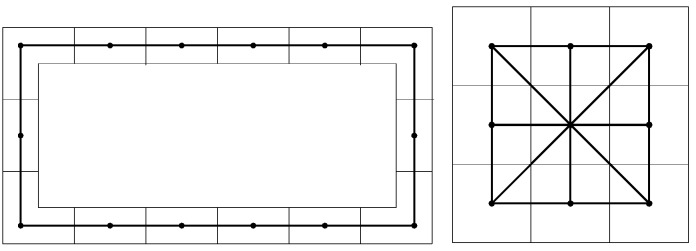
Graph structure construction.

The key idea of constructing a graph structure is to represent the indoor environment using a graph {G=(V,E)}, where V are vertices defined at each survey point during 3D modeling of the indoor environment, and E are edges that connect different segments of the 3D model if there is a direct access from one segment to another. The corresponded graph structure for a corridor area is shown in Figure 7 on the left. For a small room, we scan the entire room by standing in the middle and rotating the scanning system 360°. Thus, the room will be represented by a single vertex in the graph structure. For a large open space, the graph structure is shown in Figure 7 on the right.

A vertex $V_{i}$ encodes the 3D color point cloud $PT_{V_{i}}$, WiFi received signal strength $RSS_{V_{i}}$, and position $POS_{V_{i}}$. These attributes are stored in an object array, $A_{i}=[PT_{V_{i}},RSS_{V_{i}},POS_{V_{i}}]$. An edge $E_{ij}=\{V_{i},V_{j}\}$ connects two vertices, $V_{i}$ and $V_{j}$. The edges act as constraints for the motion choices, since the user in vertex $V_{i}$ can only directly access vertex $V_{j}$ if there is an edge $E_{ij}=\{V_{i},V_{j}\}$ between them.

By introducing a graph as the data structure, we are able to restrict the user movement and predict the user location at the next moment. Since the user can only move around connected vertices, we will only have limited amount of candidate vertices for the next move. If the space involved in a vertex is small enough, finding the user’s location is approximated as locating the vertex. This is similar to grid-based localization. However, the smaller the grid is, the higher the computational cost will be. In practice, we choose the grid size as the survey area covered during each 3D scan. Thus, locating the vertex gives a coarse estimation of the user’s location. A finer positioning within the vertex is achieved using particle filtering. The use of the graph structure also increases the system’s robustness. In the case of crowded environments, where the sensor signal fidelity may not be reliable, the constraints in the graph can help detect a sensor failure if the prediction based on the sensor’s measurement violates the constraints.

### 2.3. Gaussian Process Modeling of WiFi Received Signal Strength

A Gaussian process (GP) essentially estimates a posterior probability distribution over functions from training data (details can be found in [36]). We will give a brief introduction here.

Let us first define a function, $f(x_{*})$, be the posterior distribution that makes predictions for all possible inputs $x_{*}$. Additionally, we have $D=\{(x_{i},y_{i})|i=1,...,n\}$, which is a set of training samples consisting of n observations drawing from a noisy process, $y_{i}=f(x_{i}+\varepsilon )$, where each $x_{i}$ is an input sample in ${ℜ}^{d}$, and each $y_{i}$ is a target value in ℜ. ε is additive Gaussian noise with zero mean and variance $\sigma_{n}^{2}$. For notational convenience, the inputs of the training set are grouped into a d × n matrix X, and the observations $y_{i}$ are grouped into a vector y.

To estimate the posterior distribution over function $f(x_{*})$ from training dataset D, GP depends on a covariance function kernel $k(x_{p},x_{q})$, which specifies how the values at different points are correlated to each other. This kernel can be specified as any arbitrary covariance function, and we have chosen the widely-used squared exponential kernel

(4) $$k(x_{p},x_{q})=\sigma_{f}^{2}\exp(-\frac{1}{2l^{2}}|x_{p}-x_{q}{|}^{2})$$

Here, the parameters $\sigma_{f}^{2}$ and l are the signal variance and the length scale, which determine the strength of the correlation between different points.

Since we only have access to the noisy observations y instead of the true function value f(x), we must add a term to account for observation noise in the covariance function:

(5) $$\mathrm{cov}(y_{p},y_{q})=k(x_{p},x_{q})+\sigma_{n}^{2}\delta_{pq}$$

Here, $\sigma_{n}^{2}$ is the Gaussian observation noise and $\delta_{pq}$ is one if p=q or zero otherwise. For an entire set of input values X, the covariance over the corresponding observations y can be written as:

(6) $$\mathrm{cov}(y)=K+\sigma_{n}^{2}I$$

where K is the n × n covariance matrix of the input values, defined as $K[p,q]=k(x_{p},x_{q})$.

Note that the covariance between the observations is written as a function of the inputs, emphasizing the non-parametric nature of Gaussian process regression.

Now we can generate the posterior distribution over functions $p(f(x_{*})|x_{*},X,y)\sim N(\mu_{x_{*}},\sigma_{x_{*}}^{2})$ to predict the function value for any arbitrary points $x_{*}$, given the training data X and y:

The predicted mean and variance are:

(7) $$\mu_{x_{*}}=k_{*}^{T}{\left(K+\sigma_{n}^{2}I\right)}^{-1}y$$

(8) $$\sigma_{x_{*}}^{2}=k(x_{*},x_{*})-k_{*}^{T}{\left(K+\sigma_{n}^{2}I\right)}^{-1}k_{*}$$

The parameters $\sigma_{f}^{2}$, $\sigma_{n}^{2}$ and l control the smoothness of the functions, estimated by a GP, and can be learned from training data, by maximizing the log marginal likelihood of the observations. This learning process is completed offline, immediately after the training dataset is built.

To apply GP in WiFi signal strength modeling, the input values X correspond to positions, and the observations y correspond to signal strength measurements gathered at these positions. The GP posterior is estimated from a collection of signal strength measurements corresponding to their positions. Assuming independence between different access points, we estimate a GP for each access point separately.

### 2.4. Motion Dynamic Model

The motion dynamic model is defined as the user’s position changes over time, which are represented by the distance traveled and the heading movement. The built-in motion sensors in the mobile device, including an accelerometer, gyroscope and magnetometer, are used to track the user’s movement. We first take a look at the accelerometer measurement. If the user is standing still, it is expected that their mobile device will register little acceleration. Therefore, the standard deviation in the magnitude of acceleration is selected to detect the walking/stopped transition. If $\sigma_{\left|a\right|}<0.01$, it is very likely that the user is stopped. If $\sigma_{\left|a\right|}\geq 0.01$, however, it is not sufficient to ascertain that the user is walking. For example, the sudden movement of the user’s hands could result in a larger acceleration. Thus, we exploit the repetitive nature of walking [37].

**Figure 8:**
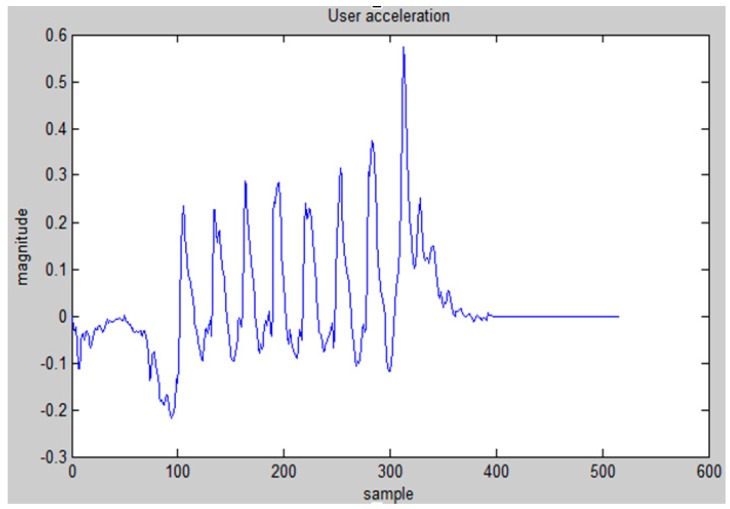
Acceleration data while walking.

Figure 8 shows the acceleration data recorded by a walking user. We can see that the acceleration data exhibits a highly repetitive pattern. This pattern arises due to the rhythmic nature of walking.

In order to determine whether the user actually enters the walking mode, we calculate the auto-correlation of the acceleration signal a(n) for lag τ at the $m^{th}$, as follow:

(9) $$\chi (m,\tau )=\frac{\sum_{k=0}^{k=\tau -1}\left[a(m+k)-\mu (m,\tau )*(a(m+k+\tau )-\mu (m+\tau ,\tau ))\right]}{\tau *\sigma (m,\tau )*\sigma (m+\tau ,\tau )}$$

where μ(k,τ) and σ(k,τ) are the mean and standard deviation of the sequence of samples from a(k) to a(k+τ−1).

If the user is walking, then the auto-correlation will spike the periodicity of the walker. We define ψ(m) as the maximum of the auto-correlation. If $\sigma_{\left|a\right|}\geq 0.01$, and ψ(m)≥0.8, then the user is very likely to be walking. Otherwise, there is no change in the motion transition state. This double threshold is able to prevent some irregular movements, for example, the movement of the user’s hand, to change the motion state.

**Figure 9:**
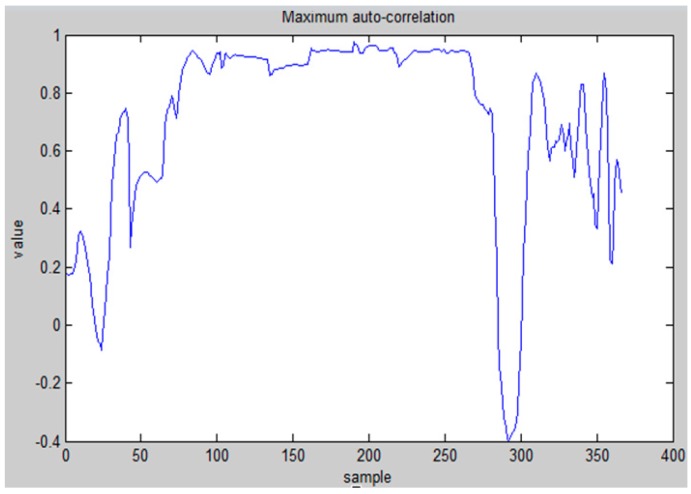
Maximum autocorrelation for step counting.

Once we have determined that the state is walking, step counting and stride estimation are performed to calculate the walking distance of the user. As shown in Figure 9, step counting is realized by dividing the duration of a sample when the maximum auto-correlation is ψ(m)≥0.8 by $\tau_{opt}$, and round up to an integer value. The $\tau_{opt}$ is determined by simply finding the most frequently occurring τ in the duration when ψ(m)≥0.8.

Because the human stride is not constant during walking, the stride size is determined by dynamically checking the acceleration sequence. We apply an empirical equation, based on [38], to estimate the stride size.

(10) $$Stride=0.98\times \sqrt[{3}]{\frac{\sum_{k=1}^{N}\left|a_{k}\right|}{N}}$$

where $a_{k}$ represents the measured acceleration and N represents the number of samples in one period of walking. The relationship between stride, period of one step, and acceleration is established through a walking test, where the tester walks with a fixed stride using ground marks.

**Figure 10:**
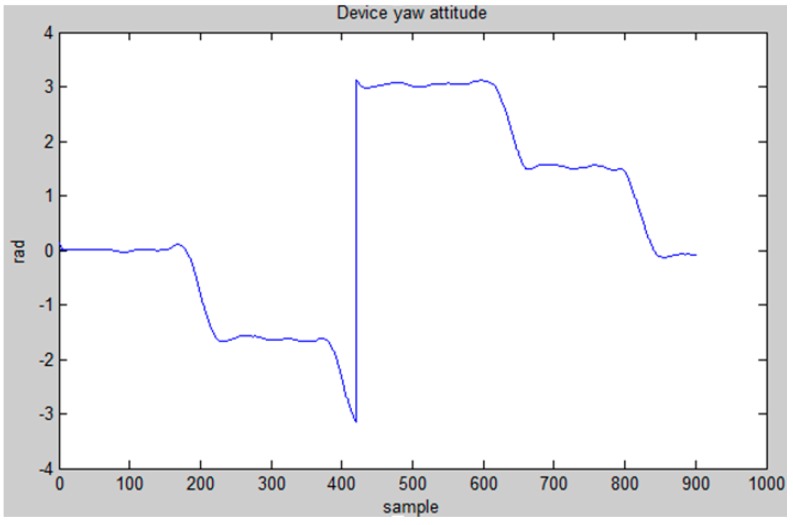
Device yaw attitude changing while walking in hallway.

In order to detect the user’s orientation, we apply a gyroscope and magnetometer sensor to detect angle change. According to reference [39], the rapid fluctuation in the sensor signals is modeled heuristically with a zero mean, white Gaussian noise. The initial orientation of the user is provided by the magnetometer sensor $\theta_{init}=\theta_{magn}+n_{magn}$, then the orientation is updated using the gyroscope $\theta_{t}=\theta_{t-1}+\theta_{gyro}+n_{gyro}$. Here, $n_{magn}$ and $n_{gyro}$ are Gaussian noise of the magnetometer and gyroscope measurements. Figure 10 shows the device yaw attitude changing while a user, holding the mobile device horizontally, is walking in a corridor.

To determine the new position, we first need to calculate the step number and stride length, and then estimate the movement as follows:

(11) x(t+1)=x(t)+((l(t)+δl(t))*cos(θ(t)+δθ(t))

(12) y(t+1)=y(t)+((l(t)+δl(t))*sin(θ(t)+δθ(t))

where l(t) and θ(t) are the estimated step length and heading direction, while δl(t) and δθ(t) are the zero mean Gaussian noises on the length and direction, respectively.

**Figure 11:**
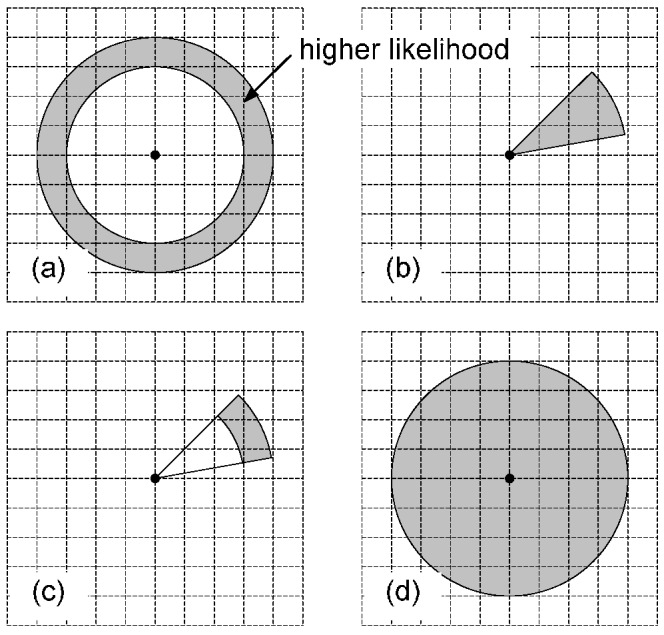
Motion dynamic model likelihood field.

The motion dynamic model fuses the information provided by different motion sensors, and indicates a higher likelihood field in particle filtering. There are four scenarios depending on the access of different kinds of motion sensors. When an accelerometer is available, the distance traveled can be estimated, based on step counting and stride length estimation. Otherwise, the distance is estimated with an empirical maximum speed, for example, 1 m/s. If a gyroscope and a magnetometer are available, the user heading is detected, if not, the heading remains unknown. Assuming an open space, the calculation of the likelihood field is shown in Figure 11. The grid points denote all possible state candidates for the next epoch, and the black dot is a state candidate of the current epoch. When only the distance traveled is measured, the likelihood field (shaded area) is located within a ring zone around the triangle, as shown in Figure 11a. The radius and width of the ring are determined, based on the measured distance and its uncertainty, respectively. If only the user’s heading is detected, and an empirical maximum speed is used to calculate a maximum walking range within a time interval, the likelihood field is shown in Figure 11b. The angle of the shaded zone is determined based on the heading and its uncertainty. If both the distance and heading are measured, the likelihood field is shown in Figure 11c. This is the case for our system, which greatly reduces the amount of particles needed for precise positioning, thus decreasing the computational complexity in particle filtering. Finally, if we have no access to any motion sensors, assuming a maximum walking range, the likelihood field is located within a whole circular area, as shown in Figure 11d.

## 3. HMM Model

**Figure 12:**
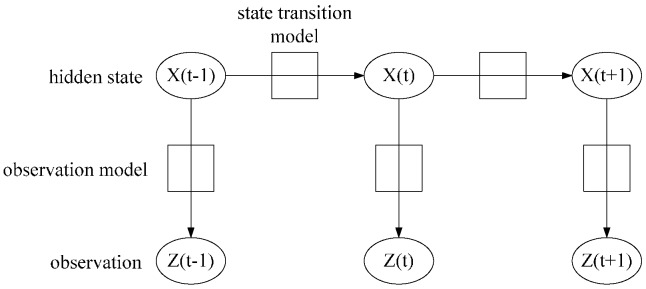
HMM model factor graph.

A general HMM characterizes a physical system with a state space model. In the problem of position tracking, the HMM model represents the temporal correlation of a user’s position and orientation. Figure 12 shows a HMM factor graph, the state X(t)={x(t),y(t),θ(t),m(t),V(t)}, where x(t),y(t),θ(t) represents the user’s position and orientation, m(t)∈(walk,stop), V(t) indicates the current vertex where the user is located in the graph structure. The state transition model $p(X_{t}|X_{t-1})$ constructed of $p(x_{t},y_{t}|x_{t-1},y_{t-1})$, $p(\theta_{t}|\theta_{t-1})$, $p(m_{t}|m_{t-1})$ and $p(V_{t}|V_{t-1})$ serve as the motion model in our algorithm. The observation model is based on the WiFi signal strength measurement, motion sensor readings, and the captured image during online tracking.

$p(m_{t}|m_{t-1})$ represents the probability of the motion state being walking or stopped given the previous motion state. It is defined as a 2 by 2 matrix, which models the preference of staying in the previous state, avoiding too-rapid changes in motion states. Moreover, we apply different matrices for different environments. This enables the system to model the fact that the user is more likely to stop in a room than in a corridor.

$p(\theta_{t}|\theta_{t-1})$ represents the probability of the current orientation $\theta_{t}$ given the previous orientation $\theta_{t-1}$. It depends on whether the user is walking or stopped, and whether he is in a hallway or an open space. As the choice is limited to two, we use binary code to represent the situation. We define Walking = 1, Stopped = 0 for the leftmost binary digit, and Hallway = 1, Open space = 0 for the rightmost binary digit in the binary representation of the situation. In total we have four situations (00, 01, 10, 11). For example, if the user is walking in a hallway, his situation code is 11. If the next motion state has been determined to be stop, the difference $\Delta \theta_{t}$ is sampled uniformly with a constant probability α~[0,2π]. Otherwise, if the next motion state is to be walking, we make a simple assumption that the user prefers to walk in a straight line, so that $\Delta \theta_{t}$ is sampled from a zero mean Gaussian distribution. If the user is in an open space, $\Delta \theta_{t}$ follows a unimodal Gaussian distribution. On the other hand, if the user is in a hallway, $\Delta \theta_{t}$ follows a bimodal Gaussian distribution, since the user has a higher chance of choosing from two opposite orientations.

$p(x_{t},y_{t}|x_{t-1},y_{t-1})$ represents the probability of the current position $\left(x_{t},y_{t}\right)$ given the previous position $\left(x_{t-1},y_{t-1}\right)$. If the previous motion state $m_{t}$ has been determined as stopped, the current position is equal to the previous one. Otherwise, the current position is updated by sampling the moving distance $d_{t}$ from a Gaussian $N(\mu ,\sigma^{2})$. Based on a simple straight motion assumption, the new position is calculated as follows:

(13) $$x_{t}=x_{t-1}+d_{t}*\cos(\theta_{t})$$

(14) $$y_{t}=y_{t-1}+d_{t}*\sin(\theta_{t})$$

$p(V_{t}|V_{t-1})$ represents the probability of the current vertex $V_{t}$ given the previous vertex $V_{t-1}$. If $V_{t}$ is in a corridor, we first calculate the walking/stopped probability. If $m_{t}=stopped$, then $V_{t}=V_{t-1}$. Otherwise, we calculate the distance that the user has travelled. For this distance, we determine whether the movement along the corridor results in a transition to another vertex, or remains within the same vertex area. The vertex transition is constrained to only two adjacent vertices. If $V_{t}$ is in an open space, it may have up to nine candidate vertices. We first sample the walking/stopped motion transition state and corresponding motion distance. Then, the vertex $V_{t}$ is determined based on a simple straight line movement.

The observation model describes the measurement likelihood of making an observation at different positions in the indoor environment. Our observation includes WiFi received signal strength, motion sensor readings, and captured image.

The WiFi signal strength measurement likelihood model uses the mean and variance of the signal at each position, calculated by Gaussian process regression.

(15) $$p(z_{t}^{WiFi}|x_{*})=\frac{1}{\sqrt{2\pi \sigma_{x_{*}}^{2}}}\exp(-\frac{{\left(z_{t}^{WiFi}-\mu_{x_{*}}\right)}^{2}}{2\sigma_{x_{*}}^{2}})$$

where $z_{t}^{WiFi}$ is the received signal strength at time t, $\mu_{x_{*}}$ and $\sigma_{x_{*}}^{2}$ are the mean and variance at position $x_{*}$, predicted using Equations (7) and (8).

The camera measurement likelihood model is computed by pairwise pixel comparison between the current view and the view of a particle [11]. In the particle filter, a given particle’s view of the environment can be projected from the 3D textured model

(16) $$p(z_{t}^{Camera}|x_{*})=\frac{\#\text{pixels in similar color}}{\text{\#total pixels}}$$

A predefined threshold determines the maximal color difference for two pixels, and a normalized color space is adopted to alleviate the effect of illumination.

The motion sensor measurement likelihood model compares the motion model $p(X_{t}|X_{t-1})$ with the motion dynamic model derived from motion sensor readings.

(17) $$p(z_{t}^{Motion}|x_{*})=\frac{1}{\sqrt{2\pi }}\exp(-\frac{|z_{t}^{Motion}-x_{*}{|}^{2}}{2})$$

where $z_{t}^{Motion}$ is the pose calculated from the motion-sensor-based motion dynamic model, $x_{*}$ is the pose estimated from the motion model defined in HMM.

## 4. Online Tracking with Particle Filter

Bayesian filtering is used to estimate the posterior state $X_{t}$ given all sensor measurements $Z_{0:t}$. Under the Markov assumption, we have the following recursive equation, which is updated whenever new sensor data become available:

$$p(X_{t}|Z_{0:t})\propto p(Z_{t}|X_{t})\int p(X_{t}|X_{t-1})p(X_{t-1}|Z_{0:t-1})dX_{t-1}$$

where $p(X_{t}|X_{t-1})$ represents the motion model, and $p(Z_{t}|X_{t})$ is the observation model.

We implement Bayesian filtering using a particle filter, which represents posterior over the state $X_{t}$ by sets $S_{t}$ of M weighted samples: $S_{t}=\{<X_{t}^{\left[m\right]},w_{t}^{\left[m\right]}>|m=1,...,M\}$. Here, each $X_{t}^{\left[m\right]}$ is a sample state, and $w_{t}^{\left[m\right]}$ is an importance weight of the state. The particle filter applies the recursive Bayesian filter update to estimate posteriors over the state space. An online tracking algorithm using a particle filter is performed according to the following steps:

(1) Particle Initialization

The initial position is calculated through the weighted K nearest neighbor (W-KNN) method. It searches for K closest matches of known positions in the WiFi received signal space from the offline-built dataset. By averaging these K position candidates with adopting the distances in signal space as weights, the initial estimated position is acquired. This initial position estimation is used as the starting point for particles.

(2) Particle propagation

Next, we apply the state transition model to guide particle propagation. During the state transition process, we observe that the particle propagation plays an important role, which represents the state transition probability. The more accurate the particles propagate towards the right position, the better the positioning performance will be. We generate new particles by sampling $X_{t}^{\left[m\right]}$ from the distribution $p(X_{t}|X_{t-1})$.

(3) Particle weight update

After particle propagation in each epoch, we weight the sample $X_{t}^{\left[m\right]}$ by the probability $p(Z_{t}|X_{t}^{\left[m\right]})$. The weight is calculated as the product of different sensor measurement likelihood function. Then the weights $w_{t}^{\left[m\right]}$ of the samples are normalized so that they sum up to 1.

(4) Particle resampling

Once the particle weights are updated, we perform importance resampling to update the particles’ state by pickinga random sample $X_{t+1}^{\left[m\right]}$ from the sample set $S_{t}$ according to the importance weight $w_{t}^{\left[m\right]}$. In resampling, the weight of each particle is treated as a probability, where this particular particle is chosen to be at the estimated position. Particles with higher weights will be picked more frequently than others. This is how the resampling is able to eliminate wrongly moved particles and correctly track the user’s position.

(5) Position estimation

After the resampling process, the estimated position is calculated as the mean of all the resampled particles’ positions. To further increase the positioning accuracy, we perform Direct Linear Transformation (DLT) [40] between the camera captured image in its current position with a projected image of the 3D model at the estimated position. The DLT parameters can be obtained using least square method. To solve for DLT parameters, we need at least six correspondences. The correspondences can easily be obtained using the SIFT or SURF feature matching technique. The matching process frequently contains “outliers”, therefore, we apply the well-known RANSAC [41] algorithm to filter out the “outliers”. After getting the DLT parameters, the camera position can be calculated by solving for the projection matrix. This process is able to refine the position estimation, but due to its high computational cost, we only invoke it in certain key vertices that are predefined in the graph structure, for example, at the corridor corner. The user can also invoke the process at their wish when they require better positioning performance. The process will also wake up after a time period T to correct the estimation error. Table 1 gives a pseudo code of the particle filter algorithm.

**Table 1:** Pseudocode of particle filter.

${\left\{X_{t}^{\left[m\right]},w_{t}^{\left[m\right]}\right\}}_{m=1}^{M}=PF\left[{\left\{X_{t-1}^{\left[m\right]},w_{t-1}^{\left[m\right]}\right\}}_{m=1}^{M},Z_{t}\right]$		
	Initialization $\left[{\left\{X_{t=0}^{\left[m\right]},w_{t=0}^{\left[m\right]}\right\}}_{m=1}^{M}\right]$	
	FOR m=1:M	
		Particle propagation $X_{t}^{\left[m\right]}\sim p\left(X_{t}|X_{t-1}^{\left[m\right]}\right)$
		Update weight using observation $w_{t}^{\left[m\right]}=p\left(Z_{t}|X_{t}^{\left[m\right]}\right)$
	ENDFOR	
	Normalize weights to $\sum_{m=1}^{M}w_{t}^{\left[m\right]}=1$	
	${\left\{X_{t}^{\left[m\right]},w_{t}^{\left[m\right]}\right\}}_{m=1}^{M}=\text{Resample}\left[{\left\{X_{t}^{\left[m\right]},w_{t}^{\left[m\right]}\right\}}_{m=1}^{M}\right]$	

## 5. Implementation on iOS Platform and Experimental Analysis

**Figure 13:**
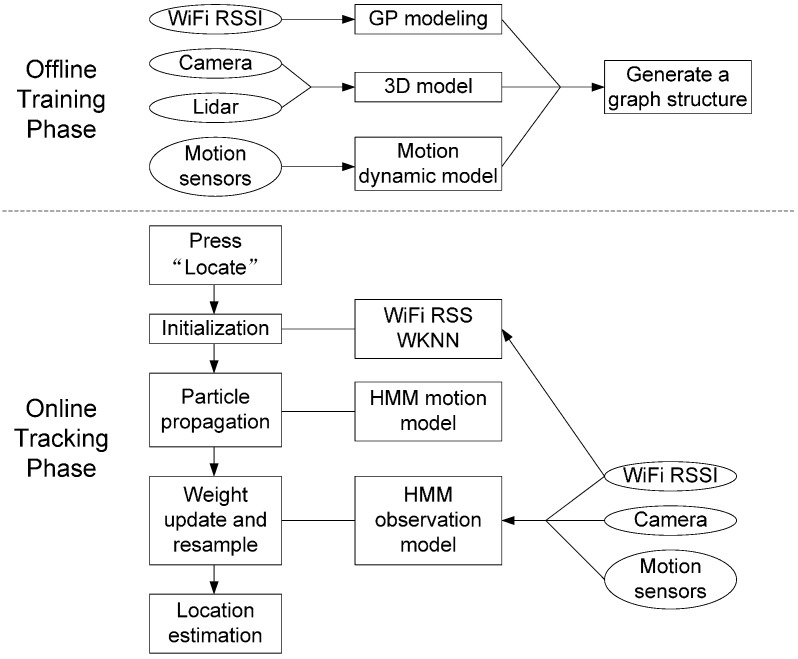
System workflow.

We realize the indoor positioning algorithm on the iOS platform and build an app to test the system performance. The system workflow is shown in Figure 13. During the offline training phase, WiFi received signal strength, color images, point cloud, and motion signals, including user acceleration, device attitude, and rotation rate, are recorded along the entire scenario. A detailed 3D model of the indoor environment is generated by fusing color images from the camera and point cloud from LiDAR. Then, we apply Gaussian process modeling to generate a signal strength map of the WiFi RF fingerprints. The WiFi signal strength map is overlapped with the 3D model and we divide the 3D model into different segments according to our survey points. Each segment is encoded in the vertices of a graph structure. We store the graph structure in the mobile device for the online tracking phase. Once we press the “Locate” button, the mobile device starts scanning the WiFi received signal strength from all the access points it can detect. Particles are initialized through weighted the K nearest neighbor method. After the initial distribution, particles start to propagate under the guidance of the HMM motion model. Every 5 s, the particles are resampled using the HMM observation model. Through particle filtering, we are able to locate the user and track the user’s movement in real time.

**Figure 14:**
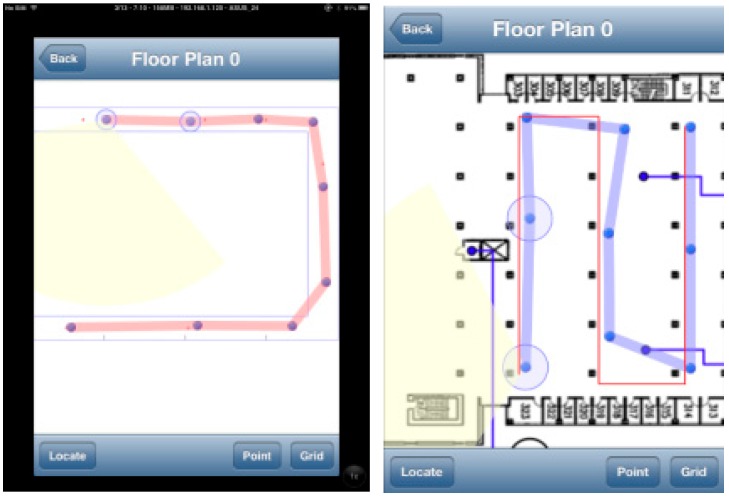
Screen shots of localization test.

The training and testing are conducted in an office building corridor area and a library’s open space. In total, we have 24 survey points in the corridor and 21 survey points in the library. Positioning tests are conducted on a predefined path. The average length of the path is about 90 m in the library’s open space and 100 m in the corridor area. The position update is performed every 5 s, which means the particle filtering step can be completed in 5 s. The memory usage is under 40 MB RAMsince the iOS app will be forced to shut down if it exceeds this limit. During the traverse on the path, we measured the error distance between the estimated position and the ground truth position. The ground truth position is based on manual annotation of waypoints. Whenever the tester reaches a waypoint, the timestamp is recorded and the estimated position with the actual one are compared. Figure 14 shows screenshots of the real time test results in the corridor and the library.

**Figure 15:**
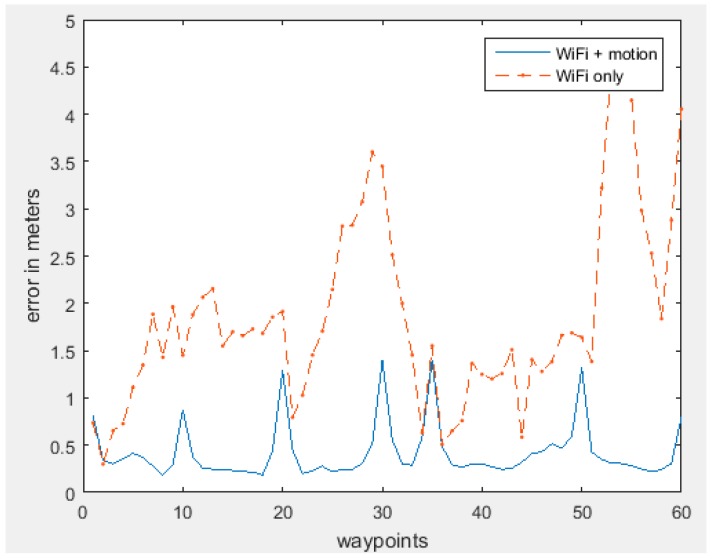
Error in each waypoint.

Figure 15 illustrates the positioning error in each waypoint. By including the motion sensors, the error has dropped significantly compared to using only the WiFi based positioning method.

**Figure 16:**
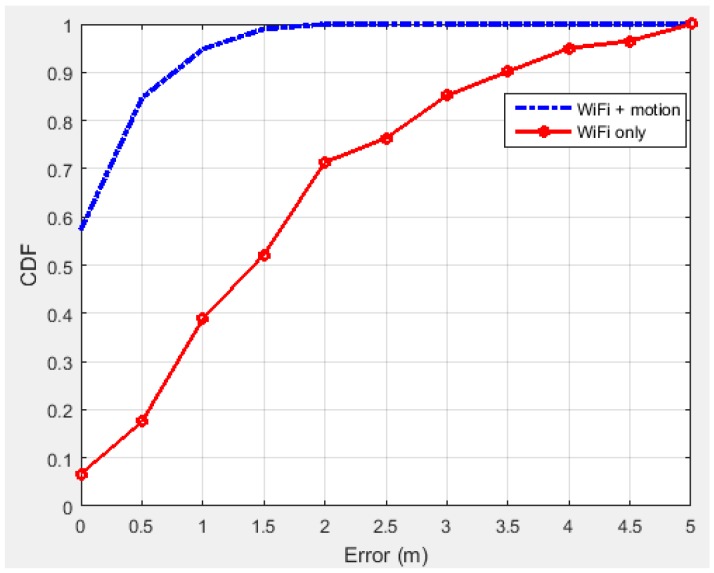
Cumulative distribution function (CDF) of error.

To gain insight into the positioning error distribution, Figure 16 presents the cumulative probabilities of the positioning errors of the different cases. The comparison further shows that the motion dynamic model greatly increases positioning accuracy.

Table 2 compares the error mean, root mean square (RMS) error, and maximum error in different scenarios.

**Table 2:** Location accuracy comparison.

Error	Error Mean	RMS	Maximum
WiFi RSSI	1.85 m	2.10 m	4.78 m
WiFi + Motion sensors	0.42 m	0.51 m	1.41 m

As we can see from Table 2, the incorporation of motion sensors with WiFi RSSI has greatly reduced the positioning error. Due to the implementation issue on the iOS device, we have not included the visual sensors in the real time test. The visual sensors are applied separately. After the real time test is done, we capture the images at all the waypoints and correct the estimated position. Table 3 shows the correction results.

**Table 3:** Visual sensor correction on localization results.

Error	Error Mean	RMS	Maximum
WiFi + Motion sensors	0.42 m	0.51 m	1.41 m
Visual sensor correction	0.10 m	0.23 m	0.66 m

Table 3 shows that the visual sensors could further improve the positioning accuracy. Overall, the positioning performance has proved that our iOS application is a robust, accurate, highly-integrated indoor positioning system. However, we have not implemented various state-of-the-art indoor positioning methods in the literature, due to the difficulty in realizing them on a mobile platform. The state-of-the-art method of a WiFi signal strength based method using Gaussian process is discussed in [4,5], we implemented this on the iOS platform. Moreover, we introduce a framework based on HMM to effectively fuse the WiFi information with motion sensor and visual sensor information on the mobile platform in order to improve system performance.

## 6. Conclusions

In this paper, we have demonstrated an indoor localization system based on a graph structure and multimodal particle filtering technique. The implementation on the iOS platform, and the test in a real world situation proved that our application is a reliable indoor localization system. To the best of our knowledge, this is the first iOS app delivering such accurate, highly integrated indoor localization system on a small mobile device. Based on our system, many position-aware applications will be able to function properly indoors, providing more convenient service to people’s daily lives.

In the future, we will focus on fusing the visual sensors on board into our real time localization system. We believe the key technology for future localization systems lies in how to efficiently fuse the information provided by various sensors. We are also looking to integrate our system into other platforms, for example, a vehicular infotainment platform, so that we have access to more sensor information and can function both indoors and outdoors.
